# Recognizing the Benefits of Pre-/Probiotics in Metabolic Syndrome and Type 2 Diabetes Mellitus Considering the Influence of *Akkermansia muciniphila* as a Key Gut Bacterium

**DOI:** 10.3390/microorganisms9030618

**Published:** 2021-03-17

**Authors:** Raluca Anca Corb Aron, Areha Abid, Cosmin Mihai Vesa, Aurelia Cristina Nechifor, Tapan Behl, Timea Claudia Ghitea, Mihai Alexandru Munteanu, Ovidiu Fratila, Felicia Liana Andronie-Cioara, Mirela Marioara Toma, Simona Bungau

**Affiliations:** 1 Department of Preclinical Disciplines, Faculty of Medicine and Pharmacy, University of Oradea, 410073 Oradea, Romania; raluca14@yahoo.com (R.A.C.A.); v_cosmin_15@yahoo.com (C.M.V.); 2 Department of Food Science, Faculty of Agricultural and Food Sciences, University of Debrecen, 4032 Debrecen, Hungary; areha.abid786@gmail.com; 3 Department of Analytical Chemistry, Faculty of Applied Chemistry and Materials Science, University Politehnica of Bucharest, 011061 Bucharest, Romania; aureliacristinanechifor@gmail.com; 4 Department of Pharmacology, Chitkara College of Pharmacy, Chitkara University, Punjab 140401, India; tapanbehl31@gmail.com; 5 Department of Pharmacy, Faculty of Medicine and Pharmacy, University of Oradea, 410028 Oradea, Romania; timea.ghitea@csud.uoradea.ro (T.C.G.); mire.toma@yahoo.com (M.M.T.); 6 Department of Medical Disciplines, Faculty of Medicine and Pharmacy, University of Oradea, 410073 Oradea, Romania; mihaimunteanual@yahoo.com (M.A.M.); ovidiufr@yahoo.co.uk (O.F.); 7 Department of Psycho-Neuroscience and Recovery, Faculty of Medicine and Pharmacy, University of Oradea, 410073 Oradea, Romania; felicia_cioara@yahoo.com

**Keywords:** prebiotics, probiotics, metabolic syndrome, type 2 diabetes mellitus, *Akkermansia muciniphila*, gut bacterium

## Abstract

Metabolic syndrome (MetS) and type 2 diabetes mellitus (T2DM) are diseases that can be influenced by the structure of gut microbiota, whose improvement is often neglected in metabolic pathology. This review highlights the following main aspects: the relationship between probiotics/gut microbes with the pathogenesis of MetS, the particular positive roles of *Akkermansia muciniphila* supplementation in the onset of MetS, and the interaction between dietary polyphenols (prebiotics) with gut microbiota. Therefore, an extensive and in-depth analysis of the often-neglected correlation between gut microbiota and chronic metabolic diseases was conducted, considering that this topic continues to fascinate and stimulate researchers through the discovery of novel strains and their beneficial properties.

## 1. Introduction

Metabolic syndrome (MetS) is a combination of risk factors, such as overweight/obesity, hypertension, and imbalances in the metabolism of lipids and carbohydrates. All components of MetS are well-known risk factors when it comes to developing type 2 diabetes mellitus (T2DM), heart diseases, and atherosclerosis. As a consequence of its pathophysiological association with other cardiovascular risks, this chronic condition is one of the main contributors to the prevalence of the disease. As obesity is a precursor of MetS, the maintenance and regulation of MetS will be important in the treatment of obesity by physical activity (e.g., exercises), lifestyle improvements (counseling), caloric restriction menu, reduction of weight by medication, or through weight loss surgery. Strategies such as exercise and behavioral improvement involve firm discipline of the mind and are difficult to follow. In comparison, calorie-limited diets in obese children have shown to be less efficient. The latest pharmacological drugs often determine adverse side-effects and high treatment costs [1].

Several researchers have suggested various nutritional solutions based on probiotic or prebiotic approaches, following a close association between food, gut microbiota, and MetS pathophysiology.

Prebiotics are a peculiar type of dietary fiber with health benefits that not only selectively administer live microbes but are also invoking alterations in the host microbial environment [2]. They can be found in a variety of sources, such are non-digestible oligosaccharides, non-digestible sugars, non-refined wheat, non-refined barley, soy, crude oats, breast milk, and inulin sources (e.g., chicory roots and Jerusalem artichoke) [3]. Prebiotics used as non-digestible food additives have a favorable effect on the host by selectively inducing a small number of colon microorganisms to develop and/or to be enabled [4]. Inulin-based fructose oligomers or galactic oligosaccharides are the majority of prebiotics [5]. Pullulan has long been used for food additives as a possible prebiotic compound [6]. Pullulan is a maltotriosis α-1.6-related polymer and is separated from the *Aureobasidium pullulan* fungus [7]. It is also considered a non-digestible carbohydrate since it has a high molecular weight and has sluggish hydrolysis of α-amylase and glucoamylase [8]. Pullulan fermented via microbiota has previously been documented to modify the composition of the intestinal microbiota [9]. The FAO and WHO have described probiotics as non-pathogenic living microorganisms, which guarantee the proper health of the host when properly used in foods or food supplements [10].

Probiotics are derived from multiple ways, such as diverse natural habitats, human intestinal microbiota, and food [3,11]. The prophylactic and therapeutic actions of some pre-/probiotics in numerous gut-related disorders are partially mediated via modification or the function of the microbiota. Considering previous definitions, probiotics acts “by contributing to [the host’s] intestinal microbial balance” [12] or “by improving the properties of the indigenous microflora” [13]. Taking into account those mentioned so far, in the current consensual form of the definition of probiotics, their effects are considered to be mediated both by microbiota and by other mechanisms. Thus, probiotic microorganisms can function via numerous pathways, including modulation of immune function; interaction with the resident microbiota; host interface; formation of organic acids, enzymes, or other antimicrobial compounds; and improving the quality of the intestinal barrier, etc.

On the other hand, prebiotics are considered to be substrates through which host microorganisms selectively confer a health benefit, their actions including mineral absorption, defense against pathogens, metabolic effects and regulation of satiety, bowel function, immune modulation, etc. The use of pre-/probiotics has been demonstrated by credible and pertinent evaluations of their effectiveness, but it should be mentioned that not all tested products have been validated. The most important target here can be considered the recommendation of these supplements (clearly based on scientific evidence) only by health professionals [14].

Research has shown that the gut microbiota is one of the host’s most significant environmental influences and establishes dynamic interactions with the hosts over their entire existence. The metabolic role of intestinal microbiota is also crucial to biochemicals in the body that save energy, produce energy-efficient compounds, and produce vitamins and other essential nutrients [15]. In addition, the intestinal microbiology protects its host from pathogenic inflammation and regulates the innate and acquired immunity [16,17]. Researchers have recently reported imbalances in intestinal microbiota, disease sensitivity, immune deficiencies, and, most specifically, obesity and resistance to insulin [18].

Other studies have shown that the prevalence and treatment of diabetes mellitus (DM), in particular T2DM, requires the use of the host microbiome. Dysbiosis has a clear association between T2D and the gut microbiota (GM) of T2D patients with opportunistic pathogens, particularly the butyrate that generates the bacteria, decreasing the number of beneficial microbes. The incoherence of gut microbiota (GM) is considerable. Improving intestinal health can help control diabetic problems and can determine more DM-related health issues from arising [19,20].

*Akkermansia muciniphila* (*A. muciniphila*) is a new highly promising probiotic. It colonizes the mucosal layer in the intestines and modulates the basal metabolism. The association between *A. muciniphila* and obesity is consistent. A number of animal models and human trials have shown the causal positive effect of *A. muciniphila* therapy on obesity, this probiotic being an enhancer actor in the metabolism of organism and having considerable potential for treating obesity-related metabolism, as well as related to the treatment including newly medicinal agents or compounds [21,22]. In this regard, a good example are the results of Shang et al., illustrating the beneficial effects of *Akkermansia* on MetS during the treatment of the disease by fucoidan [21].

The purpose of this in-depth analysis, of a topic that may seem exhausted at first glance, is to provide a very complex and complete overview regarding the relationship between probiotics/gut microbes with the pathogenesis of MetS, the roles of *A. muciniphila* in the onset of MetS, and the interaction between dietary polyphenols (prebiotics) with gut microbiota. The present paper thus consists of extremely concentrated and useful referenced information for any future works in the field. The reason we selected the current subject was the increasing prevalence of MS and its associated diseases, considering also the fact that numerous therapies are not controlled from the point of view of objectives attainment; therefore, additional measures are needed. Furthermore, supplementation with pre-/probiotics is an easy way to give to the patient another modality to control or at least ameliorate his condition and improve his quality of life.

## 2. Methodology

**Figure 1:**
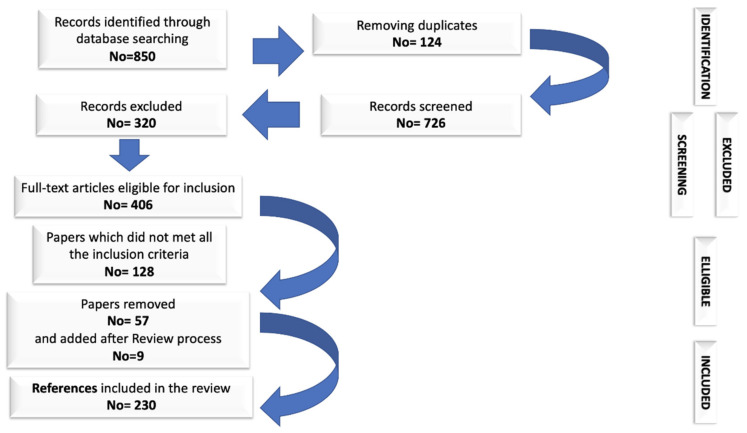
Flow chart of the selection process of the bibliographic sources included in this article.

This review involved an exhaustive research of the literature and included the selection of scientific published data addressing the topic of supplementation with pre-/probiotics in MetS and T2DM. To capture all the details relevant to the research, there was no restriction on the publication date of the selected articles, being included both recent and older works. Moreover, for the purpose mentioned above, the most relevant medical and biological data bases (PubMed/MEDLINE, EMBASE, Science Direct, etc.) for articles published until February 2021 were searched, using the representative keywords (mentioned at the beginning of this paper) in the topic or their combinations, in order to find the main data/results/papers in the field. The flow chart describing the selection process of the articles included in this research is presented in Figure 1. Were excluded the publications that were not on the topic approached in this paper and those that were not in English.

## 3. The Relationship between Probiotics/Gut Microbes with the Pathogenesis of Metabolic Syndrome

### 3.1. Intestinal Microbiota and the Link to MetS

The connection between the composition of intestinal microbiome and metabolic diseases, including obesity and diabetes, was seen in recent studies focused on large-scale sequencing of the 16S rRNA, quantitative real-time PCR (qRT-PCR), in situ fluorescing hybridization (FISH), high-throughput technology pyrosequencing, and deoxy-ribonucleic acid (DNA) bar codes. Researchers demonstrated that obesity may cause the intestinal microbiota composition to change both in mice and people. In contrast with their fat counterparts, lean laboratory animals (i.e., rats, mice and pigs) have a larger amount of *Bacteroidetes* spp., where *Firmicutes* are prevalent [23,24,25,26,27]. Therefore, in comparison to lean subjects, a decreased ratio of *Bacteroidetes/Firmicutes* was identified. There is, however, a lack of coherence between studies. Low microbial gene richness has recently been hypothesized as a good marker for MetS [28,29]. In addition, an abnormal gut microbiota is able to induce sub-acute systemic inflammation, insulin tolerance, and increased risk of CVD through bacterial exposure pathways, namely, bacterial lipopolysaccharides (LPS) [30].

Owing to the mixture of genetic and environmental causes, the prevalence of obesity, diabetes, and MetS is attributed to gastrointestinal microbiota. There are several indications that obesity and related intestinal microbiota metabolic disorders are associated with low-grade systemic inflammation [31,32]. The treatment of metabolic disorders and of obesity-related endotoxemia is important in preventing intestinal microbiota dysbiosis and maintaining the function of the epithelial barrier of the intestine [33]. Metabolic endotoxemia is the pathological disorder attributed to low-grade LPS (endotoxin) plasma elevation from the intestines to the bloodstream [34]. Studies have shown that this endotoxin exacerbates chronic metabolic disease pathogenesis and is common in people with DM, dyslipidemia, insulin tolerance, and obesity in subclinical inflammatory processes [35].

#### 3.1.1. Influence of Intestinal Microbiota Dysbiosis

The cause of dysbiosis is related to the loss of usual functions of the microbiota generating many metabolism disorders [36]. A fat-rich diet can also affect the dysbiosis process, resulting in increased serum lipopolysaccharide (LPS) and dysfunctions of the intestinal barrier [37]. In comparison, dysbiosis can aggravate the still unknown pathogenesis of chronic inflammatory disease [38].

Some bacterial cell components have an impact on the immunomodulatory function of the lymphoid tissue in the increased inflammatory phase. Researchers suggest that the translocation and signaling of antigens via the intestinal mucosal barrier are activated by cell membrane components, including peptidoglycans and LPS. A pro-inflammatory function is accomplished by binding LPS and peptidoglycan to toll-like receptor 2 (TLR4) and by activating the immune system in a cascade reaction with the oligomerization domains of nuclear oligotides (NOD) [39,40].

The LPS serum levels are double in obese, diabetic, or extremely fat people, as a result of reduced intestinal barrier resilience, increased development of chylomicron during the digestive phase, and a reduction in the function of the alkaline phosphatasis, which causes the cleavage of this endotoxin. This repeated sensitivity of MetS to serum LPS has also caused MetS association with the innate immune system, assisted by the use of the protein LPS (LBP) and CD14 co-receptor [41,42].

In the intestinal microbiota and the innate immune system, MetS production is interactive. The latest actions are aimed at creating a specific diet to reconstruct and/or strengthen the immune system underlying the intestinal homeostasis of microbiota [43]. In addition, the unique strain properties of gut microbiota have been shown by laboratory models for the attenuation of some chronic infectious immune responses [43].

While the bowel microbiota of adults is constant, food, genotypical/epigenetic combination and immune-metabolic function may determine adjustments. The signaling pathways between the host and microbiome, including different groups of ligands, are used as essential effectors for modification, as stated by Moran and Shanahan in 2014 [44,45]. β-cell syntheses, which control insulin and glucose homeostasis, are enhanced by the involvement of inflammatory biomarkers in diabetes-induced oxidative and terminal stresses [46].

If the structure of the intestinal microbiota becomes more intricate and contagious, more modifications are seen in the macrophage M2 to M1 adiposis environment, which can contribute to the development of MetS [47].

#### 3.1.2. Influence of Dietary Composition

Although the intestinal microbiome responds to major caloric intake changes, numerous experiments suggest that it is most vulnerable to dietary composition [48,49]. In a human trial, volunteers had either a plant-based (cereal, fruit, legumes) or animal-based (meats, eggs, cheeses) diet for five consecutive days. After these five days, their microbial populations underwent major behavioral shifts. Participants whose diet focused on animal products witnessed a bloom of bile-resistant microbes (*Alistipes* spp., *Bilophila* spp., and *Bacteroides* spp.) and a reduction of fiber-fermented bacteria [48]. Additional experiments have shown microbial exposure to seeds, fiber forms, and food contaminants in dietary fats [50,51,52,53].

Particularly interesting are dietary fats, since certain acids have antimicrobial activity, but this feature is calculated by the number of carbons and double C=C-bonds, their position, and orientation [54]. Caesar also found that the proinflammatory *Bilophila wadsworthia* has bloomed in rats who have been fed a lard diet, while *Lactobacillus* and *A. muciniphila* have bloomed in the diet based on fish oil [55]. An increase in the number of *A. muciniphila* was adversely connected to obesity, T2DM with medication, and elevated BP [56,57,58].

Recently, in overweight/obese insulin-resistant people, the first clinical trial involving oral *A. muciniphila* supplementation was carried out. The randomized, double-blind combination study was conducted for 3 months and revealed an increase in insulin sensibility and reduction in insulinemia and overall plasma cholesterol. Therefore, it may also be part of the solution, even though the interaction between the diet and the microbiome implies the progression of obesity [59].

An impaired microbiota is further involved in promoting the risk of cardiovascular disease. Studies performed on antibiotic and germ-free mice indicate an important role of gut microbiota in transforming nutrient phosphatidylcholine in trimethylamine N-oxide molecule (TMAO) [60,61]. In this direction, microbes cleave dietary choline through the hepatic flavin mono oxygenize 3 (FMO3) to form TMAO from animal products, which is oxidized, in return, by the liver. The levels of circulating TMAO in patients with atherosclerosis were substantially higher relative to the stable controls [61]. In recent human research, vegan and omnivores who have been confronted with a supplementing choline diet have demonstrated a dose-dependent rise in TMAO in the circulation. After treatment with large spectral antibiotics, the prothrombotic phenotype is absolutely removed, which clearly indicates that the microbiome has an important role in this phenomenon [62].

#### 3.1.3. Influence of Inflammation

Although a consistently low inflammation is not a distinctive cause for MetS, it is a recognized element in the etiopathology of obesity/insulin resistance and is thus closely related to MetS metabolism. The important function of intestinal permeability in persistent low-grade inflammation renders the microbiota as a crucial part of the inflammation in metabolic deficiencies.

Inflammation represents a series of responses of the vascularized tissue to damage or infection. This is a protective survival function, but prolonged sensitivity to stimuli and immune movement in cells can be harmful. The first study of chronic low-degree inflammation with obesity and insulin resistance was documented by Hotamisligl and others in 1993. Rotary adipose tissue tolerance to insulin was achieved by TNF-α overexpression [63,64]. This survey was eventually extended to include obese people, who had elevated TNF-α mRNA as opposed to the controls in obese adipose tissue. Scientists found that metabolic cells, such as adipocytes and hepatocytes, had closely related immune systems and vessels. This proximity provides a continuous communication between the immune response and metabolism [65,66].

In fact, multiple studies found that adipose tissue in obese individuals has increased proinflammatory markers (with expanded adipocytes in mice and humans with macrophage and dead crown like structures of phagocytic macrophages) [67,68].

Inflammatory incidents contribute to an altered metabolism, frequently determined by the experimental usage of high-fat diets; nevertheless, considerable attempts are being made to clarify which causes, aside from a high-fat diet (HFD), are triggers of inflammation [69].

The function of the intracellular endocannabinoid system (ECB) is another interesting gut-centered process that can contribute to low-grade inflammatory dysmetabolism. Obesity increased the ECB levels in adipose tissue and plasma and a strong ECB synthesis stimulator for the bacterial LPS was found in this condition [70].

Pharmaceutical or genetic inhibition of the CB1 receptor cannabinoid protects against obesity, hepatic steatosis, and low-grade inflammation. In the context of monitoring the subsequent rat experiments, investigators concluded that the intestinal microbiome ECB behavior in the colon and adipose tissue (comparing the germ-free to standard mouse and analyzing the genetically engineered microbiome) was reported to be changed. The alleged role of the ECB system in healthy persons has yet to be known, but bacterial LPS can be induced and contributes to metabolic dysfunction [71].

### 3.2. Effects of Pre-/Probiotic Administration in MetS

While some clinical trials confirm the prediction that MetS is positively affected by probiotics and/or prebiotics, other experiments have shown contradictory findings, Table 1 summarizing them.

From its inception until 4 July 2019, in PubMed and Scopus, a qualitative systematic review was performed, according to the Cochrane approach, as well as a thorough literature search for randomized, controlled experiments. Nine clinical trials were finally reviewed according to our inclusion criteria, equal to six randomized control trials (RCTs).

#### 3.2.1. Effects of Probiotics Alone

In some trials, the use of probiotics in patients with MetS has increased the index of body fat, BP, lipid profile, and glucose metabolism. In inflammatory biomarkers, probiotics also have a beneficial effect on molecule 1 (sVCAM-1), 6 (IL-6), α factor of necrosis (TNF-α), endothelial vascular factor of growth (VEGF), and thrombomodulin. The admission of probiotic compounds in patients with MetS leads to discreet changes in some clinical features of MetS and a decrease in inflammatory biomarkers, considering the diversity of the published reports [102].

In a Swedish analysis of the HFD-fed mice supplemented with *L. plantarum*, the lack of any reaction, specifically to the intravenous glucose-tolerance test, was related to the administration route that could not induce glucagon-like peptide-1 and release insulin. After collection from 426 strains, *L. plantarum* was used in a Korean sample as well [103].

The decline in insulin resistance parameters was associated to the cell reports of increased insulin signaling and inflammatory genes. *L. casei* interference improved the concentration of *Lactobacillus* and *Bifidobacterium* and decreased *Clostridium* in hyper insulinemic fructose-fed mice. The reduction of GLP-2 induced by *Bacteroides fragilis* was related to decreased insulinemia, while performing oral glucose tolerance tests (OGTT) [104]; moreover, some advantages were also found in liver function genes and gene expressions.

*L. coryniformis* administration with HFD-fed mice has caused vascular benefits and could be correlated with improvements in the composition of microbiota and intestinal permeability with reduced endotoxemia [105]. In research conducted in Taiwan, the use of lyophilized and “live” *L. reuteri* supplements increased *Lactobacillus* and lowered the abundance of pathogens, which helped improve the intestinal barrier. Weight gain, lipid profile change, hepatic steatosis, as well as inflammation and insulin resistance-related gene expressions decreased [106]. *L. reuteri* has also been used with the Aryl hydrocarbon receptor antimicrobial agonist MS in a French analysis. The addition improves glucose metabolism and liver alanine transaminase. Added synthesis increased glucose and hepatic alanine transaminase levels. In a Korean published paper, HFD-fed mice to whom *L. sakei* was administered as well, gained both weight and epididymal fat mass, by decreasing the inflammation and increasing the intestinal barrier (increased genetic expressions of tight-joint protein) [107]. Another two experiments in diabetic mice demonstrated similar decreases in insulin tolerance, TNF-α, and IL-6 at a dosage of 10^9^ CFU pioglitazone and separate doses of *L. casei* [108]. The increased concentration of *Lactobacillus* and *Bifidobacterium* and SCFA has been linked to a potential rise in glucose homeostasis. Traditionally, probiotic activity stopped pancreatic islets from degeneration and/or regeneration.

An analysis comparing metformin, vildagliptin, and three *L. rhamnosus* strains (GG, MTCC5690, MTCC5689) increased the insulin tolerance curve areas and OGTT, with the exception of the HFD-MTCC5690 treatment [109]. Probiotics enhanced bowel permeability, increased adiponectin, and reduced inflammatory markers. Metabolic advantages gained through probiotics are similar to metformin and vildagliptin.

An Indian study on diabetic mice revealed greater results on the glucose metabolism, lipid profiles, and oxidation stress markers with *L. rhamnosus* NCDC17 compared to *L. rhamnosus* GG supplements. Moreover, adiponectin, pro-glucagon, and prohormone genes (TNF-α and IL-6) were found as being inflamed [110]. HFD-fed mice received *Lactobacillus rhamnosus* in order to minimize the abundance of *Bilophila wadsworthy* associated with MS [111]. Additionally, the *B. wadsworthia* increased in accordance with the effects of glucose metabolism, but the probiotic only enhanced the implications of HFD in the event that these bacteria were in high abundance.

In a Japanese study with *L. gasseri* in fermented milk, the complementation increased the production of insulin by reducing pancreatic/systemic inflammation [112]. *L. paracasei* was tested in a study for the advantages of glucose and lipid metabolism in obese rats and for many indicators of renal function and inflammation [113]. A research showing a decline in weight gain, visceral fat accumulation, and inflammation in combination with an increase in glucose and lipids studied the role of sterilized *B. longum* in obese mouse [114]. By comparing *B. lactis* and *B. longum* supplements, improved productivity of the former in terms of acetate and metabolic benefits was revealed [115]. For two doses of *B. breve* in HFD-induced mice, weight and visceral fat were decreased and genetic expressions decreased [116]. The authors also suggested that the probiotic could enhance healthy commensal bacteria to prevent endotoxemia and inflammation in an experiment with *B. animalis* [117]. The benefits of *C. butyricum* supplementation were complemented by increased intestinal and antimicrobial effects, supplementing glucose and lipid metabolism [118].

#### 3.2.2. Effects of Associated Pre-/Probiotics and Synbiotics

Four studies have compared pro-/prebiotics and synbiotics (which are combination between pro- and prebiotics). Many of these have been xylo-oligosaccharide (XOS), inulin, and fructo-oligosaccharide and prebiotics (FOS). First, dysbiosis and inflammation were assessed in rats that were obese, with improved synbiotic results [119]. The groups treated also reduced the *Firmicutes* spp.-to-LPS ratio and raised the concentration of *Bifidobacteria* spp. in the prebiotic culture. The investigators concluded that prenatal, probiotic, and synbiotic products contribute to a decrease in endotoxemia and inflammation. The second research study investigated the link between cognitive function, *L. paracasei*, XOS, and the gut–brain axis in obese rats, which are shielded against insulin [120]. The glucose and lipid metabolism improved with prebiotics, probiotics, and synbiotic interventions, but the adiposity decreased with the XOS and synbiotic. Both products were beneficial, improving brain mitochondrial function and hippocampal plasticity, decreasing also the microglial activation. The third research study explored the association between psychiatric disorders and oxidative stress following addition or mixture of inulin or *L. plantarum* [121].

All therapy improves oxidative stress and psychoactive effects (depressive and anxiety-like behaviors). During the fourth study, in which HFD-fed mice were given *L. paracasei*, FOS, or their combination, the impacts on NAFLD were assessed [122]. Benefits during intraperitoneal GTT were considered as follows: increased gut barriers, decreasing LPS levels, and successful insulin signaling pathways were found in all of the treated populations. Steatosis improved, as well as dyslipidemia and inflammatory disorders.

#### 3.2.3. Additional Results Obtained with Probiotics of Various Strains

In one study, *Lactobacillus* spp. and *Bifidobacterium* spp. were combined, and two other *Lactobacillus* spp. plus one *Bifidobacterium* spp. strains were added [123,124]; the first two mediated beneficial modifications in microbiota structure, bodily adiposity, insulin tolerance, and dyslipidemia in HFD-induced obese mouse. In comparison to the isolated effect of each probiotic on the composition, *B. animalis* had the most pronounced preventive effect on the structure [123]. Another Brazilian trial demonstrated the regulation of the composition of microbiota and increased intestinal permeability, endotoxemia, and inflammation in a mixture of three probiotics. The treatment also included a drop in insulin and leptin hypothalamic tolerance, which influenced the eating pattern (reduced food intake and weight gain) [124].

## 4. The Roles of *A. muciniphila* in the Onset of Metabolic Syndrome

The only Gram-negative emblematic *Verrucomicrobia* that is widespread in human intestinal mucosa is *A. muciniphila* [125]. Gene sequence analyses revealed that there are multiple gene candidates for mucin encoding and the single chromosome is contained in 2176 genes with a 55.8% GC content after the MucT type strain of *A. muciniphila*, as reported by Derrien (ATCC BAA-835 1/4 CIP107961T) [126,127]. This non-motile, oval-formed microorganism is purely anaerobic and with chemical organotrophic material that can withstand low levels of oxygen. *A. muciniphila* may create mucin-degrading enzymes and use mucin in the mucosal layer of the epithelium as a source of carbon and nitrogen. *A. muciniphila* splits these compounds into acetic and propionic compounds, releasing sulfate [128,129]. *A. muciniphila* also is 3 to 5% of the total gut microbiome population in healthy adult humans based on an analysis of its distinctive 16SrRNA signature, although this amount differs by several factors. In stable human beings, *A. muciniphila* has been closely linked to age. Its colonization starts at a young age and ranges between 5.0 and 8.8 log cells/g in a year equivalent to the adult stage but decreases in the elderly [130,131]. In comparison, in patients with metabolic disorders, *A. muciniphila* and a mucosal pathology varied, and the incidence of appendicitis and IBD was reversely associated with this [132]. In addition, a negative association of intestinal *A. muciniphila* with diabetes, obesity, and others MetS has been shown [133].

Dual control of *A. muciniphila* and metabolic disorders has demonstrated that both *A. muciniphila* excess and supplementation can have an effect on the host body. The anatomy of *A. muciniphila* can also be affected. The dissemination of *A. muciniphila* through early vancomycin therapy to the early intestinal colonization could help regulate the progression of autoimmune diabetes [134]. In an initial analysis, *A. muciniphila* was recognized as a potential new therapeutic agent for obese patients. Most studies have shown the advantages of using *A. muciniphila* in metabolic and obesity disorders for prevention and progress. T2DM is characterized by a lower *A. muciniphila* abundance, low inflammation, and an intestinal permeability disease [135]. The degree of *A. muciniphila* development can be used to determine the metabolic state of the body, for instance glucose homeostasis, serum lipids, and human adipocyte distribution. The reason for associating *A. muciniphila* with the production of obesity is not yet entirely explained. We have therefore analyzed the most recent studies into *A. muciniphila’s* function in obesity and learned more about its effects on the distinctive changes in the expression of pathways in metabolic homeostasis [136].

### 4.1. A. muciniphila and Obesity: Evidence from Mouse Models

All of the previous studies found a correlation between the caloric intake of *A. muciniphila* and its abundance. Prebiotic administration in high-fat diet mice eliminated the metabolic endotoxemia that characterizes the compromised MetS present among obese participants, decreased the overall fat mass, and decreased body weight [137]. These findings have in fact been strongly linked to an abundance of *A. muciniphila* [138]. The reduction of *A. muciniphila* and inflammatory markers were crucial in all circulatory parameters (i.e., glucose, insulin, leptin, and triglycerides), with 118 of the 13 genes implicated in the fatty oxidation, production, and oxidation of positive ties, whereas *Bifidobacterium* spp. were important. Three inflammatory features, leptin, and only two genes involved in the oxidation of fatty compounds are positively and negatively related.

Schneeberger et al. documented the inversely related levels of *A. muciniphila* in mice with inflammatory markers, lipid synthesis and insulin tolerance, cardiovascular risk, and adiposity markers in their plasma. After six consecutive weeks of HFD administration, the main effects were body weight gain and adiposity in mice. The results therefore suggest that an HFD particularly affects the gut bacteria and show that the abundance of *A. muciniphila* is decreasing steadily with sustained dietary care in mice. This bacterium also reduces, showing a causal impact, the disease development before the initiation of metabolic changes [139]. In comparison, the abundance of *A. muciniphila* increases and decreases in mice fed a diet rich in fish oil and lard, with greater regulation of the intestinal barrier system and less inflammatory tissue, which can be converted into germless receiving mice [55]. Other authors also found that the gut barrier dysfunction, weight, and fat gain of HFD-fed mice can be decreased at the same time with *A. muciniphila* [140,141]. The relation between age and *A. muciniphila* was finally identified in mice, as the intestinal level of the bacterium is lower in older mice. An HFD improves the adipose tissue and intestinal microbiological composition considerably compared to aging [139].

### 4.2. A. muciniphila and Obesity: Evidence from Human Studies

Emerging research assessed the correlation between *A. muciniphila* intestinal abundance and human body weight. There are proofs that these two variables have an opposite correlation [142,143]. The numbers of *Bifidobacterium* spp. and *A. muciniphila* and the number of *Staphylococcus* spp., *Enterobacteriaceae* spp., and *Escherichia coli* were found to decrease in overweight pregnancy. They were analyzed by quantitative real-time PCR for their gut microbiota composition. The increase in overall bacteria and *Staphylococcus* was linked in the whole population with increased plasma cholesterol levels, while the increased amount of *Bacteroidites* spp. was associated with an increased level of HDL and folic acid [144].

Twenty overweight or obese children and 20 average regular weight children aged 4–5 years were evaluated by Karlsson et. al. Interestingly, in obese/oversized infants, the *A. muciniphila* levels have decreased dramatically, whereas in the same class, the Gram-negative Enterobacteriaceae concentrations were significantly higher. Bifidobacterium levels in obese/overweight children were inversely linked to alanine aminotransferase (ALT) [145]. Since diabetes and overweight are linked to increased intestinal permeability and low inflammation, endotoxemia caused by LPS is considered as one of the causative agents of obesity-related metabolic disorders. An in vitro observation that confirms the epithelial barrier role of *A. muciniphila* may provide a working hypothesis to streamline in vivo proof that links decreased fecal A. *muciniphila* with diabetes and obesity rate. This could reveal a mechanism for protecting HFD obese mice from bacterial LPS endotoxemia. No relationship was currently assessed between *A. muciniphila* and hypothalamic food intake regulation markers. According to all data, it can be concluded that *A. muciniphila* influences the reaction of humans in terms of improving inflammation, insulin resistance, and glycemia on their diet of caloric constraint [146].

### 4.3. A. muciniphila Medicinal Role in the Treatment of Metabolic Conditions

The study of *A. muciniphila* has contributed to a better understanding of its possible therapeutic effects and actions in determining certain diseases and metabolic disorders. *A. muciniphila*, in particular, tends to be involved in the development of amine butyrate and in the propionate extracellular pool. It is also important in the development of sulfide hydrogen, which can be anti-inflammatory and a powerful antioxidant [147,148]. The involvement of *A. muciniphila* in metabolism enhances prebiotic intake. Both studies performed on animals with *A. muciniphila* revealed that it decreases body weight and fat mass rise, hepatic steatosis, inflammation, cholesterol, and atherosclerosis; it also enhances insulin sensitivity and restores intestinal barrier function by influencing various factors, such as the thickness of the mucosal membrane, close attachment proteins, antimicrobial peptides, and immunity. *A. muciniphila* works in particular on the immunomodulatory involvement of a special protein called Amuc 1100 [149].

Ottman et al. recently documented the increase of glucose resistance and a decline in body weight and fat gain in mice fed an HFD relative to the untreated mice with refined recombinant Amuc 1100 protein. The critical role of *A. muciniphila* in intestinal health, particularly in metabolic immunomodulation, is evident in this scenario. In order to increase their therapeutic application to gastrointestinal disease, future studies will also need to accentuate the main role of this microorganism and of its proteins in metabolic regulation and immune modulation [150].

The clinical studies focused on the obesity implying *A. muciniphila* and its associated biomarkers are presented in Table 2.

**Figure 2:**
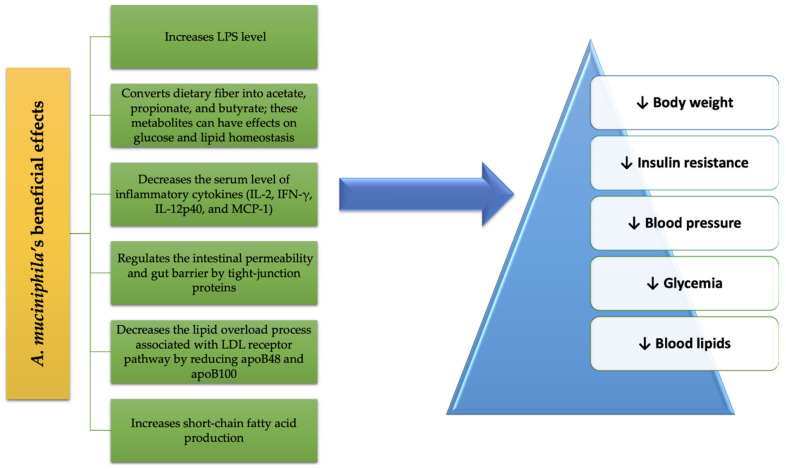
*A. muciniphila*’s benefits in MetS.

A summary of the beneficial effects of *A. muciniphila* treatment on MetS conditions is schematized in Figure 2.

## 5. The Interaction between Dietary Polyphenols (Prebiotics) with Gut Microbiota

Dietary polyphenols are considered naturally existing substances in plants or obtained from foods (i.e., cereals, coffee, fruit, tea, vegetables, and wine [167,168,169]. It is estimated that the small intestine consumes just 5–10% of the gross intake of polyphenol. Residual polyphenols, along with conjugates excreted into bile in the intestinal lumen, may accumulate in large intestinal lumps to a millimolar stage, where they are subjected to the enzymatic activity of the intestinal microbial group [170].

The gut microbiota and polyphenols have two major complementary relationships. On the other hand, after ingestion, dietary polyphenols are subject to a dynamic metabolism, associated with human and microbial enzymes, which contributes to the production of a broad variety of circulating and excreted metabolites and catabolic items. Polyphenols and their metabolites may on the contrary affect and cause a modification of the structure of gut microbiota via numerous interactions. Some studies support the possibility of modifying and producing variations in the populations of microflora by providing prebiotic effects and anti-pathogenic intestinal flora after dietary phenolic substances reach the gut microbiota, along with aromatic metabolites produced [165,166,167]. Human beings regard the biochemical transformation of dietary polyphenols as a significant concern. A minimal volume of dietary polyphenols is consumed by the small intestine, often after conjugation reactions such as de-glycosylation [166,170]. After absorption into the small bowel, polyphenolic compounds of lower complexity can undergo biotransformation in the enterocyte and then in the liver cell, by step I (oxidation, reduction and hydrolyses). These transformations produce a metabolite of water-soluble conjugates (glucuronide, sulfates, and methyl derivatives) that are rapidly released into the systemic circulation for subsequent organ drainage and urine excretion. The polyphenolic backbone of 90–95% unabsorbed polyphenols comprises colonic bacterial enzymes in the vast intestinal system that manufactures metabolites, which results in various physiological effects [171].

Colonic microflora could turn polyphenols into bioactive compounds that could affect the intestinal ecology and human health. The recommended concentrations will alter the composition of the gut microflora by inhibiting particular bacterial groups, as studies in humans and animal have revealed. Others will prosper in the open niche of the ecosystem [172].

Besides processing the derived food, the gut microbiota may perform a variety of bio-transformations of the polyphenols that enter the colon and influence their intake and bio disposability [172]. Specifically, some phenols in the metabolism of *Clostridium* spp. and *Eubacterium* spp. were reported to include isoflavone (daytzine), flavonol (quercetin and kaempferol), flavanone (naringenin and iso-xanthumol), and flavan-3 oil (catechin and epicatechin) [173]. Polyphenol colonic fermentation produces a wide range of biotransformation ingredients, but most of them are derived from phenyl, phenyl propionic, phenyl-butyric, valerical, valerolactone, phloroglucinol, and di-benzo-pyran urolithin A or urolithin B [174]. Microbiota degrade the parent polyphenolics in a variety of products, including phenolic acids such as 3-hydroxyphenyl acid, from flavanol routines synthesis (found in tomatoes, for example). The health benefits of these indirect and final materials arise with the assumption that certain bioavailability factors may be clarified for many biological effects traditionally linked to polyphenols (relative to that of the parent compounds) [175]. Phenolic acids can be found both in the plasma and urine after a meal, but some are activated by mammalian enzyme mechanisms (for example protein catabolism), which make it difficult to understand the plasma and urine levels of phenolic acid. A greater inhibition of anti-platelet aggregation has been shown by 3,4-dihydroxy (3,4DHPAA), one of the key intermedial metabolites of rutile-catabatic acid [176], as well as of secretion of monocytes of the proinflammatory cytokines TNF-a and IL-6 [177] than by the parent compound. It has also been demonstrated that phenolic acids suppress protein glycation [178]. However, most phenolic acid bioactivity research experiments are still based on in vitro models and more in vivo evidence is needed.

The absorption rate of polyphenol is very low and up to 90% of these substances linger in the colon [179]. They are metabolized through bacterial esterase, glucosidase, demethylation, dihydroxylation, and decarboxylation processes, resulting in smaller metabolites, several of which can be absorbed by the intestinal mucosa, such as phenolic acids and short-chain fatty acids. It is important to know that the microbial bioconversion potential of each organism influences and impacts their bioavailability on the final metabolites produced. In reality, since individuals have their own unique intestinal microbiota synthetic signature that can permit a fingerprint comparison, it can modulate the effect of polyphenol on host welfare by human intestinal microbiota composition [173]. On the other hand, the modulating microbiota of the intestinal ecosystem can be influenced by polyphenols and their metabolites. Certain phenolic compounds with bacteriostatic or bactericidal behavior were considered potential antimicrobial agents. In addition, they can also serve as inhibitors of infection-causing bacteria within intestinal and urinary tract cells, suggesting that such phenolic compounds are capable of being used as antimicrobial agents against human pathogens [180].

### 5.1. Polyphenol Transformation via Intestinal Microbiota

Polyphenols are not well absorbed by GI proximity and tissue responses to high-molecular PPs are now impaired by the metabolism of PP-rich food (such as tea, wine, chocolate, and fruit) [181]. Glycosidic bond cleavage and heterocyclic column breakdown, for example, are related to the biotransformation of polyphenols (PPs) (e.g., anthocyanins) via GM [182]. This biotransformation could be accomplished by different reactions, such as oxidation, hydroxylating, dehydrogenation, decarboxylation, isomerization, glycosylation, methylating, etc. Buddleoside flavonoids were transformed into aglycon acetins by human intestinal microflora, resulting in the transformation of metabolites into methylated and hydroxylated by-products. This research has defined the ability to transform flavonoids extensively, by just four bacterial species, such as *Escherichia* sp. 4, *Escherichia* sp. 34, *Enterococcus* sp. 45, and then *Bacillum* sp. 46 [183].

Maya-Apaza et al. suggested that the formation of large metabolites of 4-hydroxybenzol-propionic acids was the outcome of human GM formation during polyphenol fermentation (cyanidin-glycosyl-rutinoside, quercetin-rutinoside, chlorogenic, and neochlorogenic acids) [184]. The latest laboratory studies have shown that human genetic materials (caffeic acid, 3,4-dihydroxybenzoic acid, 2,4,6-tryhydroxybenzoic acid, coumarin, p-coumaric acid, ferulic acid, 2,4,6-trihydroxybenzaldehyde, and gallic acid) are produced by human GM fermentation in blackberry anthocyanin extract. The metabolites that were produced after 6 h of fermentation were caused by total cyanidin-3-glucoside degradation [185]. Recent research recorded the transformation of *Lactobacillus acidophilus*, genetically engineered microbes, into aglycons which can further be changed or used directly in dietary plant glycosides by other bacterial organisms [186].

It can be concluded that polyphenol degradation metabolites are predominantly responsible for the host’s health benefits based on the available evidence.

### 5.2. Modulatory Impact of Polyphenols on Intestinal Microbiota

A proper diet, which includes fruits and vegetables, leads to achieving the best health. Polyphenols (PPs), owing to the potent antioxidant role, have positive health effects, and are associated with fruit and vegetables [187]. The improvements in the bioavailability of PPs and their metabolites are also influencing the structure of the gut microbiota. Considering the data from in vitro studies, both the animal and human trials show, however, that selected PPs could serve as “prebiotics” and modify the ecology of the intestinal microbiota, thereby affecting host health. PPs also play a vital role in providing health outcomes, such as weight loss by GM change and host/microbe interactions [188]. A recent study by Sun et al. found that tea PPs obtained from the samples *Bifidobacterium* spp., *Lactobacillus* spp., and *Enterococcus* spp. have a significant impact on the gut microbiome [189]. The collections have increased SCFA efficacy and minimized the distribution of *Prevotella, Bacteroides*, and *Clostridium histolyticum*. In a study that shows the effect of black tea and red wine/grape extract on human gut microbiota, Kemperman et al. used the stimulated intestinal microbial environment, SHIME [190]. According to their study, the growth of *Bifidobacteria*, *B. coccoides*, *Victivallis*, and *Anaeraeroglobus* was improved by *Klebsiella* spp., *Enterococci* spp., and *Akkermansia* spp. simultaneously. The red wine/grape extract also encouraged the production of *Alistipes* spp., *Cloacibacillus* spp., *Victivallis* spp., and *Akkermansia* spp., and reduced the development of *Bacterioidites* spp., *Bifidobacterium* spp., *B. coccoides*, *Subdoligranulum* spp., and *Anaeroblobus* spp. It has been shown that red wine extract fermentation decreases the in vitro growing of *Clostridium histolyticum*, a harmful bacterial species in GM [191]. Similar findings were presented in a study carried out with human volunteers and in relation to *Clostridium histolyticum* development accompanied by red wine consumption [192]. On the other hand, Cueva and others note that the bacterial growth of the intestines can be greatly impaired by in vitro fermentation of the grape-derived flavan-3-ols [193].

### 5.3. Benefits of Polyphenols in MetS Patients

Many studies have shown that fruit-containing PPs play an important role in reducing the complications related to obesity [194,195,196]. An increasing part of the literature also points to a role of PPs in defending against diabetes and related complications by genetically modified organisms. The high prevalence of intestinal *Bacteroidetes* due to the improved potential for glycan degradation is caused by a PP-rich diet. Researchers thus hypothesized that microbiota modulation may contribute to the anti-obesity and antidiabetic mechanism through polyphenols [197]. The administration of polyphenol-rich cranberry extract (CE) avoided a high fructose–high sucrose dietary weight gain and visceral obesity development in mice. CE also decreased the production of triglycerides and prevented liver inflammation, thus improving the sensibility to insulin and avoiding the development of NAFLD. Furthermore, scientists observed that the positive outcomes of CE therapy are related to increasing *Akkermansia*’s abundance [198]. The effect of dietary supplements with grape polyphenols (i.e., a decline in the ratio of *Firmicutes* to *Bacteroidetes*) was substantially modulated in HFD-fed mice [199]. The GP supplementation has also stimulated *A. muciniphila* development and protected these improvements against the harmful effects of a high-fat diet [200]. The preventive impact against obesity and related risk factors has been identified with procyanidin supplement (PS) intake. PS treatment reduced weight gain, improved dyslipidemia, and reduced energy consumption. The positive influence of PS on obesity was also beneficial related to the effect of genetically modified modulation. The 16s gene sequencing RNA study showed that PS administration significantly increased *Bacteroidetes* β-diversity, intestinal microbiota, and the *Firmicutes–Bacteroidetes* ratio. Even an increase is considered to describe obesity-driven dysbiosis in *Firmicutes/Bacteroidetes*. In addition, studies suggest that an increased pathogenesis and the progression of obesity and T2DM have also been linked with *Lachnospiraceae* spp. [201].

Everard highlighted the opportunity to improve metabolic symptoms by supplementing with *Akkermansia* spp. as a probiotic for LPS prevention and intestinal permeability reduction [141]. Li and his colleagues found sinapine polyphenol to alter the intestinal microbiota in high-fat diet-induced mice in preventing NAFLD. It is a chronic low-grade inflammatory liver condition. The administration of Sinapine reduced the ratio of Firmicutes to Bacteroidetes and increased the abundance of *Lactobacillaceae, Akkermansiaceae*, and *Blautia*. Moreover, Sinapine stimulated G protein-coupled upregulation 43 (GPR43) of short-chain fatty acid (SCFA) in order to suppress the causes of inflammation [202,203,204]. A recent research study also found that PPs decreased microbiota dysbiosis by scavenging intestinal reactive oxygen species (ROS) through gut metabolism. Grape PPs suppressed the fat-driven generation of intestinal ROS and thereby decreased metabolic syndrome mediated by intestinal dysbiosis. Grape PPs stimulated healthy anaerobic intestinal bacteria, including *A. muciniphila*, which was linked with increased human metabolism [205]. These results revealed that dietary polyphenols may eradicate metabolic diseases by modulating intestinal microbiota positively.

### 5.4. Triggering A. muciniphila with Dietary Polyphenols

An increasing abundance of *A. muciniphila*, for instance, was correlated with antidiabetic effects of metformin in gut microbiota of obese rats in which obesity was induced through diet (DIO mice) [206,207].

A recent study has demonstrated that regular administration of polyphenol-rich cranberry extract (CE), orally, stopped weight gain over 8 weeks and enhanced many characteristics of metabolic syndrome, along with a major increase in *Akkermansia’s* abundance in DIO-mice intestinal microbiota [198]. That was the first confirmation that a fruit extract rich in polyphenol rich from fruits has a prebiotic effect on *Akkermansia*, which enhances the metabolic condition. These results have been supported by a more recent study on grape extracts, also being correlated with the prebiotic effect on *Akkermansia*, in increasing the metabolism in DIO mice [200]. In line with those observations, in addition to butter fat, the diet of mice was supplemented with California table grape powder, leading to lower adiposity and lipogenesis in conjunction with a higher trend in *Akkermansia* in intestinal microbiota [208]. Since both cranberries and grapes contain significant pro-anthocyanidin concentrations (PACs, also called condensed tannins), the unique class of polyphenols in this prebiotic operation can be considered of special significance. Cranberry PACs have also previously been correlated with increasing mucus production in mice and can provide *Akkermansia* with sufficient trophic resources [209].

Together with a propensity towards a growing degree of *Akkermansia* in gut microbiota, two new experiments have confirmed the benefits of administering flavonol-quercetin to obese rats [210]. Another study found that *Akkermansia* spp. was present in stool samples of healthy participants eating pomegranate extract (a rich source of ellagitannins) [211]; this new research being especially important, not just because it shows that human gut microbiota often has big effects on *Akkermansia* spp. when polyphenol rich extracts is called for, but also because healthy persons can benefit from the polyphenols that influence *Akkermansia* spp., which has prebiotic effects.

Interestingly enough, Roopchand et al. reported the colonic or jejunal expression of Muc2 mRNA after using Concord grape extract, type B PCs, and other polyphenols, which suggests that the *Akkermansia* niche has a direct effect. These findings, namely that polyphenols in grape juice and red wine increase *Akkermansia* spp.’s abundance, supported the hypothesis when applied to the in vitro intestine model [190].

Several clinical published data on *Akkermansia* spp. have shown an improvement in dietary obesity and are linked to a decline in weight gain, obesity, and increased glucose tolerance [206]. Live *A. muciniphila* administration has reversed obesity and metabolism by reducing adiposity, inflammation signs, insulin tolerance, and strengthening gut barrier in HFD mice [141]. Recently, the introduction of capsaicin (a dietary polyphenol) was shown to contribute to a reduction in weight gain, in mice fed with HFD, and excess of the *Akkermansia* spp., *Bacteroides* spp., and *Coprococcus* spp. [212]. Another work reveals that cranberry extract exerts positive metabolic effects by enhancing the dietary features caused by high fat/high sucrose (HFHS), which is linked to a proportional growth in *Akkermansia* spp. population [198].

The dietary replacement of an HFD with grape polyphenols contributed to dramatical improvements in the composition of the intestinal microbial population, which included a decline in the Firmicutes-to-Bacteroidetes ratio and an improvement in *A. muciniphila*. These modifications will provide some protection against the harmful effects of an HFD [200].

## 6. Pre-/Probiotic Formulations Using Nanotechnology: Applications in MetS

Emerging uses of nanotechnology [213] have recently been developed and applied in prebiotics and probiotics [214,215]. Probiotic nanotechnology is an evolving discipline that creates and allows entirely new options for the use of probiotics. In contrast with their use in medicines and pharmaceuticals, their uses in agriculture and the food industry are comparatively new. Currently, the production of nano-encapsulated probiotics is the fundamental application of probiotic nanotechnology. In order to improve flavor, texture, and consistency, the nanostructured food ingredients are created. Nanotechnology applications in producing organic food need vigilance, since their environmental and human health effects are not well established. There are currently no laws directly regulating or restricting the production of nanosized particles, primarily because of a lack of awareness of hazards [216].

### 6.1. Prebiotics Formulations

In the last few years, various forms of prebiotic formulations have gained further interest. The most favorable and most studied formulation of these prebiotics is the encapsulation into emulsions or structures dependent on nano and microparticles.

Maria et al. have therefore investigated the effect of the incorporation of numerous prebiotic fibers on the rheological and technological properties and the microstructure of an emulgated meat product (bologna). It was concluded that the stability of the released meat is improved with the inclusion of partial manhole starch and prebiotic fibers, to ensure that the provision of bologna sauce is safe [217].

Kazmierczak et al. identified a revolutionary strategy, loading these nanoparticles onto a biological carrier that delivers the treatments to a target site (a novel, nontoxic, therapeutic strain of *Salmonella typhimurium* that has been engineered as a biological delivery system hindering prostate cancer cells). Hu et al. also presented an example of nanoparticle-embodied probiotic bacteria that demonstrated an effective strategy to produce powerful and scalable DNA vaccines by covering live bacterial cells with synthetic nanoparticles [218,219]. Feher documented the use of nano-sized particles for prevention and treatment of neuroinflammation [220]. In reality, as an alternative to classical antibiotics to tackle bacterial resistance, probiotics attract the particular interest of people. Kim et al. recommended that prebiotics be produced to improve the function of probiotics by conjugating pathalic anhydride with dextran to create prebiotic formulations composed of *Pediococcus acidilactidi* [221]. They measured the cellular effects and the antimicrobial properties of the manufactured nanomaterial. Increased development of antimicrobial peptides using a self-defensing mechanism was observed for *P. acidilactidi* in phthalyl dextran nanoparticles, with an increased antimicrobial impact against both type of microorganisms—Gram (+) and Gram (−). Prebiotic phthalyl inulin nanoparticles were previously documented to boost *P. acidilactici’s* antimicrobial activities as well [222]. The increased antimicrobial activity of *L. plantarum*-treated phthalyl pullulan nanoparticles with *Escherichia coli* K99 and *Listeria* monocytogenes was also documented by Hong [223].

### 6.2. Probiotics Formulations

The nanoparticle was internalized by an energy-related and galactose-dependent conveyor mechanism in *L. plantarum* and higher amounts of plantaricin were secreted out from the nanoprobiotic produced as compared to the probiotic alone. The application of probiotic spores as a supply chain for chemotherapeutic drugs was recently proposed. In the autonomous production of nanoparticles in the gastrointestinal tract, Song et al. developed modified deoxycholic acid spores packed with doxorubicin and sorafenib [224]. Such a strategy targets the safety of medications in order to boost bioavailability in oral administration. In addition, disintegrated hydrophobic protein and hydrophilic deoxycholic acid are the source of the release, which increases the basolateral release of drugs by enhancing epithelial cells through the pathway to bile acid. Aziz identified the anticarcinogenic activity of nanoparticles of silver/*Lactobacillus rhamnosus* GG [225]. With the MTT test, the authors demonstrated that the viability of the HT-29 cell lines was substantially diminished, and apoptosis was caused by the addition of the maximum measured concentration of nanoparticles. It was observed that the synthesis of silver/*Lactobacillus rhamnosus* GG nanoparticles are cost efficient and offers a feasible biomedical nano-probiotic approach.

Fung’s work should be remembered, where the use of nanofibers to nanoencapsulate *L. acidophilus*, using 8 percent polyvinyl alcohol as a probiotic encapsulant for electrospinning technology, was suggested after investigating agro-waste-based nanofibers [226].

In Ebrahimnejad’s recent paper, chitosan is used as a probiotic bacteria to enhance the viability and survival of *L. acidophilus* nanoencapsulation towards gastrointestinal environments. Their usage as a probiotic bacterium was identified by the nanoencapsulation of probiotic bacteria [227].

### 6.3. Symbiotic Formulations

In a study of the impact of including inulin in alginate beads, Atia et al. developed an embedded oral-symbiotic supplement and observed its capacity to protect three different probiotic strains: *P. acidilactici, L. reuteri*, and *L. salivarius*. The bacteria were not found to be impaired by the encapsulation of the antimicrobial and probiotic properties [215].

A symbiotic nano-emulsion based on inulin was developed by Krithika and Preetha for enhancing probiotic stability; as a transport system for various probiotics in food products, the whey protein concentrate/inulin nano complex may be proposed [228]. Salmeron et al. published their work on the production of the fermented beverages with synbiotic properties in the field of food produced to boost human health and well-being by integrating specific and special bioactive nanoparticles, to enhance organoleptic proprieties, nutrient absorption, and the supply of bioactive nutrients and compounds [229].

## 7. Concluding Remarks and Future Perspectives

The administration of prebiotics and/or probiotics in MS is accompanied by numerous benefits in terms of improving metabolic parameters such as BMI, abdominal circumference, basal or postprandial glycemia, insulin resistance, and inflammation parameters. The quality of the microbial flora also influences numerous pathophysiological processes specific to MetS and T2DM (i.e., insulin resistance, pro-inflammatory status, regulation of blood sugar or appetite, etc.). In recent years, the attention paid to the bacterium *A. muciniphilia* is due to the observed and proven benefits in reducing body weight, with numerous studies (as highlighted in this review) supporting the need for colonization with *Akkermansia* spp. Moreover, the consumption of foods rich in polyphenols positively influences the bacterial microflora. Nanotechnology can also offer numerous solutions for overcoming the gastric barrier, as well as for achieving adequate concentrations of compounds at the intestinal level, the administration of pre-/probiotics being one of the applications of this expanding field.

Overall, several studies have shown that favorable manipulation of the probiostatic or prebiotic usage of the intestinal microbiota and the immune system will boost typical parameters of MetS. However, more properly planned animal and human experiments may reveal new information in order to justify the controversies surrounding the impact and doses of pre-/probiotics on the MetS risk factors and offer a more complete explanation of the function of the acts concerned. Specific clarified considerations include the minimum required for producing beneficial benefits, the duration of supplementation, persistence of this influence, and potential contraindications. Diverse hypotheses were newly introduced to clarify the advantages of probiosis to methyl microorganisms; however, there were substantial issues that must be considered with respect to probiotic effects and their action mechanisms regarding the strain specificities and duration of administration. Furthermore, the chemical composition of prebiotic compounds is a crucial consideration for the regulation of microorganisms of the intestinal microbiota. To explain the efficacy of pre-/probiotics on MetS prevention and management and endorse their future application of clinical procedures, scientifically randomized, placebo-controlled trials, utilizing, for example, multiple subjects, should be undertaken [230].

Pharmacists are encouraged to be familiar with pre-/probiotic products that are generally available so that they can make reasonable decisions for particular groups of patients. It is important for these drug specialists to realize when a probiotic should be prescribed, that not all strains are the same, and that certain benefits of this type of supplement are unique to each type of condition or disorder. Equipped with proper expertise, pharmacists may advise patients related to the strains or mixtures, in close correlation with the action/therapeutic advantage that the subject is looking for.

Of course, there are some additional features of a quality probiotic product, as well as a scientifically sustained benefit that must be mentioned. The following data should always be included on the probiotic label: adequate storage directions; CFU amount before date of expiry; date of expiry; details on the producer company’s contact; microbe names/genus/species/strains; and the prescribed dosage or section in compliance with clinical evidence, etc. When a probiotic is recommended, it must be well-known that not every strain is the same, that the benefits are strain-specific, so the patient must pick, related to the advantage, a scientifically proven strain. Moreover, they must select a probiotic according to scientific data, at the dosage given for that strain (or mixture of strains), and be careful when considering the following specific aspects: a high CFU count (more not being always good) and probiotic formulations of multi strains (many of them may not having clinical evidence).

## Figures and Tables

**Table 1:** Summarizing the published data related to the clinical trials considering the effects of pre-/probiotics on the associated risk factors to MetS development.

Type of the Controlled Trial	Form of the Product; Species/Active Substance; Period of Administration, Dose of Pre/Probiotic	Effects/Actions	Ref.
**Dyslipidemia**			
Randomized double-blind placebo, crossover	Lyophilized probiotic culture capsules; *Bifidobacterium animalis* ssp. lactis MB 2409 (DSM 23733) + *Bifidobacterium* MB 109 (DSM 23731) + *Bifidobacterium longum* ssp. longum BL04 (DSM 23233) (10^9^ CFU/g); 12 weeks, 1/day	↓: LDL-C, TC ↑: HDL-C	[72]
Randomized double-blind placebo	Lyophilized symbiotic capsules; *Lactobacillus acidophilus* CHO-220 (10^9^ CFU/g) + 0.2 g Inulin; 12 weeks, 4/day	↓: LDL-C, TC	[73]
**Hypercholesterolemia**			
Single-arm, open-label pilot study	Probiotic culture capsules; *Saccharomyces cerevisiae* var. boulardii CNCM I-1079 (1.4 × 10^10^ CFU); 8 weeks, 2/day	↓: RLP-P	[74]
**Hypertensive adults**			
Double-blind placebo	Fruit drink with probiotic bacteria; *Lactobacillus plantarum* DSM 15313; 1 × 10^9^ CFU/day dose; 12 weeks, 1/day	Not effective: BP parameters	[75]
	Yogurt; *Enterococcus faecium* + *Streptococcus thermophilus* 4.7 × 10^11^; 8 weeks	↓: SBP, DBP	[76]
	Sour milk; *Lactobacillus helveticus* + *Saccharomyces cerevisiae*; 7 × 10^10^; 8 weeks		[77]
	Capsules; *Lactobacillus reuteri* 5.8 × 10^9^; 8 weeks		[78]
	Cheese; *L. plantarum* 7.5 × 10^12^; 3 weeks		[79]
**Hypertensive overweight women**			
Double blind, randomized	Cheese with probiotic bacteria/*Lactobacillus casei* 01 (10^8^ CFU/g); 4 weeks, 1/day	↓: TC, LDL-C, TG, SBP, DBP ↑: HB, HDL-C, HE	[80]
**Metabolic syndrome**			
Randomized double-blind placebo	Pre/probiotic culture in yogurt/milk; *Bifidobacterium lactis* Bb-12 (10^7^ CFU/g) + 6 g inulin; 10 weeks, 2/day	↓: BFM, BFP, HOMA-IR, TG, WC, ↑: 25(OH)D, HDL-C, QUICKI	[81]
	Pro/synbiotics culture in packages; *Lactobacillus acidophilus + Bifidobacterium bifidum + Bifidobacterium lactis + Bifidobacterium longum* (1.5 × 10^9^ each); 24 weeks, 6 g/day Observation: synbiotics (containing the above-mentioned probiotics) + inulin (prebiotic)	↓: HDL-C (probiotic group)	[82]
	Probiotic culture in yogurt/milk; *Bifidobacterium lactis* Bb-12 (3.6 × 10^6^ CFU/300 g) + *Lactobacillus acidophilus* La-5 (4.4 × 10^6^ CFU/300 g); 8 weeks, 1/day	↓: BG, INS, HOMA-IR ↑: QUICKI	[83]
	Milk; *Lactobacillus casei*; 10^8^ cells/mL, 65 mL bottles × 3/day, 12 weeks	↑: High-sensitive CRP (1.86 mg/L (probiotic group) vs. −1.60 mg/L (placebo group), *p* = 0.016); LBP levels (5827 ng/mL (probiotic group) vs. −1510 ng/mL (placebo group), *p* = 0.023)	[84]
	Cheese; *Lactobacillus plantarum*; 1.5 × 10^11^ CFU/g, 50 g/day, 12 weeks	↓: BMI (−2 (probiotic group) vs. −1.6 kg/m^2^ (placebo group), *p* = 0.031	[79]
	Milk; *Lactobacillus casei*; 10^8^ cells/mL, 65 mL bottles × 3/day, 12 weeks	↓: sVCAM-1 level (−195 ng/mL (probiotic group) vs. 30 ng/mL (placebo group), *p* = 0.008 ↑: high-sensitive CRP level (1.86 mg/L (probiotic group) vs. −1.60 mg/L (placebo group), *p* = 0.002	[85]
	Milk; *Lactobacillus plantarum*; 10^7^ CFU/g, 80 mL bottles × 1/day, 12 weeks	↓: Glucose levels in FM group vs. NFM group (−10.5 (FM group) vs. −3 mg/dL (NFM group), *p* = 0.037	[86]
	Milk; *Bifidobacterium lactis*; 3.4 × 10^8^ CFU/mL, 80 mL bottle × 1/day, 6 weeks	↓: BMI variation (−1.3 (probiotic group) vs. −0.3 kg/m^2^ (control group) *p* = 0.017); TC (−15 (probiotic group) vs. 6 mg/dL (control group), *p* = 0.09); LDL-C (−17.5 (probiotic group) vs. −2 mg/dL (control group), *p* = 0.08)	[87]
Randomized double-blind placebo, cluster cross-over	Probiotic culture in fermented milk; *Bifidobacterium animalis* ssp. lactis HN019; 45 days, 1/day, 2.72 × 10^10^ CFU/mL	↓: BMI, TC, TNF-α, IL-6, LDL-C	[87]
**Overweight/obesity**			
Randomized double-blind placebo	Prebiotic packages; 0.29 g oligofructose/kg body weight + 0.14 g oligofructose/kg body weight; 120 days, 1/day	↓: BMI, BW, INS, HOMA-IR, LDL-C, WC	[88]
	Lyophilized probiotic culture capsules; *Lactobacillus gasseri* BNR17; 12 weeks, 2/day, 400 mg/capsule of low dose (10^9^ CFU) or high dose (10^10^ CFU);	↓: VAT (high dose), WC in both dose (high and low)	[89]
	Prebiotic packages; 2 g oligofructose; 12 weeks, 1/day	↓: BG, BW, INS	[90]
Randomized, controlled, parallel, double-blind, factorial trial	Lyophilized probiotic cultures yoghurt/capsules; *Lactobacillus acidophilus* La-5 (3 × 10^9^ CFU) + *Bifidobacterium animalis* Bb-12 (3 × 10^9^ CFU); 6 weeks, 1/day	Not effective: lipid profile, BP	[90]
**T2DM**			
Randomized double-blind placebo	Prebiotic packages; 10 g chicory inulin + oligofructose; 8 weeks, 1/day	↓: AST, ALP, DBP, FSG, HbA1c, SBP, SC	[91]
	Prebiotic packages; 10 g oligofructose + inulin; 8 weeks, 1/day	↓: BMI, DBP, IFN-γ, IL-12, WC, ↑: IL-4	[92]
	Symbiotic packages/*Lactobacillus acidophilus* (2 × 10^9^ CFU) + *Lactobacillus casei* (7 × 10^9^ CFU) + *Lactobacillus bulgaricus* (2 × 10^8^ CFU) + *Lactobacillus rhamnosus* (1.5 × 10^9^ CFU) + *Bifidobacterium breve* (3 × 10^10^ CFU) + *Bifidobacterium longum* (7 × 10^9^ CFU) + *Streptococcus thermophilus* (1.5 × 10^9^ CFU) + 100 mg oligofructose; 6 weeks, 1/day	↓: FPG ↑: HDL-C	[93]
	Symbiotic packages; *Lactobacillus* + *Bifidobacterium* (10^10^ CFU/g) + *Lactococcus* (6 × 10^10^ CFU/g) + *Propionibacterium* (3 × 10^10^ CFU/g) + *Acetobacter* (1 × 10^6^ CFU/g); 8 weeks, 1/day	↓: HOMA-IR, IL-1β, TNF-α	[94]
	Probiotic yogurt; *L. acidophilus* La-5, *B. lactis* Bb-12; 8 weeks, 300 g/day, 3.7 × 10^6^ CFU/mg for both organisms	↓: FBG, HbA1c	[95]
	Dietary supplements, *Lactobacillus reuteri* DSM 17938; 12 weeks, low dose (10^8^ CFU)/high dose (10^10^ CFU),	↓: HbA1c, insulin sensitivity index	[11]
	Probiotic fermented milk; *Lactobacillus casei, L. acidophilus,* bifidobacterial; 8 weeks, 300 mL/day	↓: FBG, HbA1c	[96]
	Probiotic bread; *Lactobacillus sporogenes;* 8 weeks, 120 g/day, 1 × 10^8^ CFU/g)	↓: FBG, insulin, HOMA-IR, HOMA–B, QUICKI	[97]
	Tablets; *Saccharomyces cerevisiae;* 12 weeks, 1800 mg/day = 6 tablets	↓: FBG, HbA1c, HOMA- IR, QUICKI,	[98]
Randomized double-blind placebo crossover	Synbiotic packages; *Lactobacillus sporogenes* (10^7^ CFU/g) + 0.05 g β-carotene + 0.1 g inulin; 6 weeks, 3/day	↓: INS, HOMA-B, HOMA-IR, TC/HDL-C ratio, TG, VLDL-C	[99]
Randomized triple-blind placebo	Prebiotic packages; 10 g Inulin; 8 weeks, 1/day	↓: FSG, HbA1c, HOMA-IR, hs-CRP, INS, TNF-α, LPS	[100]
**T2DM + CHD**			
Randomized double-blind placebo	Probiotics culture and Se packages; 200 μg/day Se + 8 × 10^9^ CFU/g probiotic (*Lactobacillus acidophilus + Lactobacillus reuteri + Lactobacillus fermentum + Bifidobacterium bifidum* (2 × 10^9^ CFU/g each); 12 weeks, 1/day	↓: FPG, HOMA-IR (probiotic + Se), hs-CRP, INS, TC, TG, VLDL-C, ↑: GSH, NO, TAC, (co-supplementation)	[101]

Legend: ↑—increase;↓—reduction/decrease; 25(OH)D—25-hydroxyvitamin D; ALP—alkaline phosphatase; AST—aspartate aminotransferase; AV—abdominal visceral; BFM—body fat mass; BFP—body fat percentage; BG—blood glucose; BMI—body mass index measure; BP—blood pressure; BW—body weight; CFU—colony forming unit; CHD—coronary heart disease; DBP—diastolic blood pressure; FPG—fasting plasma glucose; FSG—fasting serum/blood glucose; GPA—glutathione peroxidase activities; GSH—total glutathione; HB—hemoglobin; HbA1c—glycated hemoglobin; HDL-C—high-density lipoprotein cholesterol; HE—hematocrit; HOMA-B—homeostasis model assessment index-β-cell; HOMA-IR—homeostasis model assessment index-insulin resistance; hs-CRP—high-sensitive C-reactive protein; IFN-γ—interferons-γ; IL-1β—interleukin-1β; IL-2—interleukin-2; IL-4—Interleukin-4; INS—insulin; LDL-C—low-density lipoprotein cholesterol; LPS—lipopolysaccharides; NO—nitric oxide; QUICKI—quantitative insulin sensitivity check index; RLP-P—remnant lipoprotein particle; SBP—systolic blood pressure; SC—serum calcium; Se—selenium; SFA—subcutaneous fat areas; T2DM—type 2 diabetes mellitus; TAC—total antioxidant capacity; TAS—total antioxidant status; TC—total cholesterol; TG—triglycerides; TNF-α—tumor necrosis factor alpha; VAT—visceral adipose tissue; VLDL-C—very low-density lipoprotein cholesterol; WC—waist circumference.

**Table 2:** Clinical studies of obesity implying *A. muciniphila* and its associated biomarkers.

Patients Included in the Study/Condition/Period of Study	Observations	Results	Ref.
81/T2DM/3 months	A reduced-energy diet	Consumption of *A. muciniphila* according to dietary portfolio improved levels and strengthened glycemic regulation, dyslipidemia, and inflammation.	[151]
60/overweight + obese diabetes/45 days	600 mg butyrate + 10 g inulin/day powder, or placebo	Insulin + butyrate supplementation may increase *A. muciniphila*, and butyrate lowers the expression of TNF-alpha mRNA, hs-CRP, MDA, and DBP.	[152]
28 men/obese + metabolic syndrome/35 days	1 g resveratrol orally, 2/day or placebo	Resveratrol increases homeostasis of glucose and *A. muciniphila* abundance.	[153]
134 prediabetes 134 healthy controls	Observation	There was a strong decline in the concentration of the mucin-degrading bacterium *A. muciniphila* in prediabetes.	[154]
49/overweight + obese/12 weeks	Calorie restriction for 6 weeks	The large amounts of *A. muciniphila* increased the distribution of fasting plasma glucose, plasma triglycerides, and body fat.	[136]
43/hypercholesterolemic 19 healthy controls/2 years	27 patients with Atorvastatin treatment	Treatment with atorvastatin improved the amount of *A. muciniphila*.	[155]
70 female patients/T2DM 70 healthy females.	Observation	Decreased *A. muciniphila* was linked with fasting blood/urinary glucose.	[156]
16 infants/obese mothers 256 infants/normal mothers as control	Observation	Prevalence of *A. muciniphila* was lower in control infants with normal mothers.	[144,157,158]
28/diabetes 84 healthy controls	Metformin	Patients with diabetes who obtained metformin have a higher relative abundance of *A. muciniphila* vs. healthy controls.	[159]
13/morbidly obese patients/12 months	Roux-en-Y gastric bypass (RYGB)	Within the first 3 months, RYGB modified the relative abundances of 31 species, including *A. muciniphila*. This increase in abundance can be continued for 9 months.	[160]
53 women/obesity	Observation	There were 140 metagenomic species associated with metabolic markers, including *A. muciniphila*.	[140]
21/T2DM/12 months	Duodenal-jejunal by-pass surgery medical care	In the surgery control group, the amount of gut *A. muciniphila* increased.	[57,161]
32/overweight + obese insulin-resistant/3 months	Oral supplementation of 10^10^ *A. muciniphila* bacteria, live or pasteurized	For liver dysfunction and inflammation, *A. muciniphila* decreased body weight and decreased the levels of the related blood markers, although the overall composition of the gut microbiota remained unchanged.	[59]
21/alcoholic steatohepatitis 16/healthy controls	Observation	The concentration of fecal *A. muciniphila* vs. healthy controls (indirectly related to the incidence of hepatic disease) was diminished in ASH patients.	[162]
13/overweight adults/7 weeks	Interventional, fasting (1 week), followed by probiotic intake (6 weeks) Samples: feces, T1 = before fasting, T2 = during fasting, T3 = 6 weeks after probiotic intervention	In comparison to fasting (T2), the concentration of *A. muciniphila* was higher before fasting (T1) than after probiotic action (T3)	[137]
3c/normal weight 3/morbid obesity 3/after gastric bypass surgery	Interventional, gastric bypass	Reduced quantity of *A. muciniphila* in obese subjects (who obtained an elevated quantity of *A. muciniphila* after gastric bypass) vs. average weight subjects	[163]
11/colorectal cancer 10/healthy	Observational	Increased *A. muciniphila* level in colorectal cancer patients vs. in healthy subjects	[164]
71/T2DM 74/healthy controls		Increased *A. muciniphila* abundance in T2DM patients’ feces, vs. in healthy controls patients	[165]
53/obese women		No correlation of *A. muciniphila* abundance with dyslipidemia/insulin resistance	[140]
Children (4–5-year-old) 20/normal weight 20/overweight 20/obesity		Reduced *A. muciniphila* abundance in overweight/obese vs. in normal weight children	[145]
Pre-diabetes/newly diagnosed T2DM: 44/normal 64/pre-diabetes 13/T2DM		Reduced *A. muciniphila* abundance in patients with pre-diabetes/T2DM vs. subjects with regular glucose resistance	[56]
23/autistic children		Reduced *A. muciniphila* abundance in autistic children’ feces	[131]
Overweight/obese adults 10/normal weight 10/overweight 10/obesity		No correlation of *A. muciniphila* level had with the value of BMI	[166]

Legend: BMI—body mass index; DPB—diastolic blood pressure; T2DM—type 2 diabetes mellitus.
